# Influence of Water on the Adsorption Sites of Cycline Antibiotics onto Hydroxyapatite Surfaces

**DOI:** 10.1021/acs.langmuir.5c03448

**Published:** 2025-09-29

**Authors:** R. Soria-Martínez, Alexandre Malta Rossi

**Affiliations:** Centro Brasileiro de Pesquisas Físicas, Rio de Janeiro 22290-180, Brazil

## Abstract

Hydroxyapatite (HA) functionalized nanoparticles are particularly important in bone and tooth regeneration. This study continues previous work aimed at understanding the interaction of cycline antibiotics with different HA surfaces, now incorporating the effect of hydration on adsorption energies as calculated using density functional theory. The strength of these interactions was analyzed through the quantum theory of atoms in molecules (QTAIM). The adsorption isotherm of nearly stoichiometric HA followed Langmuir–Freundlich kinetics. Minocycline exhibited higher adsorption energy than doxycycline and tetracycline. The nonhydrated adsorption behavior of minocycline is compared to earlier results for doxycycline and tetracycline, while this study also evaluates adsorption on hydrated HA surfaces for all three molecules. Minocycline displays more stable adsorption than the others, in agreement with experimental findings. Our results reveal that the influence of water cannot be neglected: although it does not prevent cycline-HA interactions, it can stabilize previously unstable configurations through hydrogen bonding between cyclines and water molecules. QTAIM analysis indicates that a combination of electrostatic interactions and medium-to-strong covalent character governs the adsorption mechanism.

## Graphical abstract

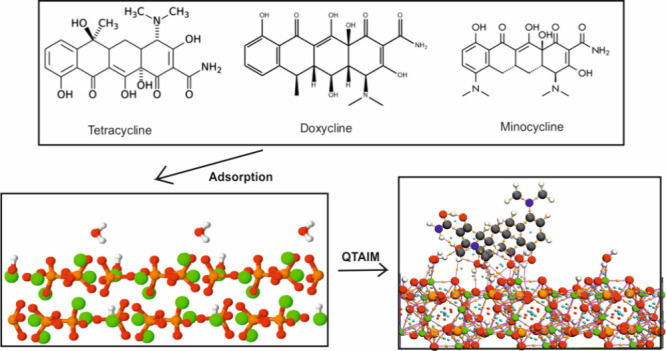

## Introduction

Hydroxyapatite (HA) is one of the most widely used biomaterials for bone regeneration due to its biocompatibility and osteoconductivity.^1–3^ When functionalized with drugs, its surface can act as a delivery system in treatments for tumors and inflammatory conditions. A key challenge in developing effective HA-based delivery systems is optimizing drug binding to the HA surface while controlling release kinetics. Broad-spectrum antibiotics from the cycline family, such as tetracycline (TC), minocycline (MINO), and doxycycline (DOX), have been loaded onto HA and tested as delivery systems for treating oral and long bone infections.^4–9^ Microbiological evaluations have confirmed the antibacterial efficacy of HA and metal-substituted HA loaded with these antibiotics. Preclinical studies have further shown that DOX and MINO can enhance bone regeneration.^10–13^

Despite these promising results, controlling long-term release kinetics remains the primary obstacle to developing effective and safe HA-based delivery platforms. In this context, a deeper understanding of the chemical interactions between antibiotics and HA nanoparticle surface sites is urgently needed. Experimental studies have investigated the adsorption kinetics and release profiles of these antibiotics using HA-based nanoparticles and nanocomposites.^14–19^ The HA crystal structure allows for ionic substitutions and has the ability to bind to growth factors, chemotherapeutic agents, and antibiotics, which supports its use as a therapeutic and osteogenic agent in clinical procedures.^20–22^ HA bone grafts are often impregnated with antibiotics and antimicrobial agents to prevent bacterial proliferation and treat infections associated with bone loss.^23–29^

However, few studies have combined experimental and theoretical approaches to investigate the adsorption sites in detail. In a previous study published elsewhere, we described the adsorption sites of DOX and TC on nonhydrated 010 and 001 HA surfaces.^30^ We evaluated the experimental surfaces characterized by Ospina et al.,^31^ in which the 010 surface can be divided into two distinct terminations: PO_4_-rich and OH-rich, referring to phosphate-rich and phosphate-deficient surfaces, respectively. Surface energy analysis revealed that the 001 surface is the most stable, while the 010-OH is the least stable. Electrostatic potential mapping of these surfaces revealed electrophilic and nucleophilic regions, which provided initial insights into potential interaction sites. Nucleophilic regions were associated with the OH and Ca atoms, while electrophilic regions were located around the phosphate groups. Comparison between theoretical adsorption energies and experimental adsorption results indicated that DOX had greater affinity for HA surfaces than TC. The adsorption kinetics of nearly stoichiometric HA (Ca_1__0_(PO_4_)_6_(OH)_2_) followed Langmuir–Freundlich behavior. Atomistic DFT simulations revealed that both molecules preferred the 010-PO_4_ surface in their most stable nonhydrated configurations.

Water plays a fundamental role in physiological and biochemical environments and is essential in the biomineralization process. The water/HA interface has been extensively investigated using molecular dynamics and DFT approaches. Wei et al.^32^ used classical molecular dynamics to show that strong noncovalent interactions at the HA surface create ordered water layers, reducing water mobility. De Leeuw studied the interaction of water with HA, particularly when OH^–^ groups are substituted by F^–^.^33,34^ Additionally, Corno et al.^35–39^ conducted detailed DFT investigations into water adsorption on the (001) and (010) HA surfaces.

In this study, we use the same HA models as in the previous work and obtain experimental adsorption isotherms for MINO. Adsorption energies of MINO on nonhydrated HA surfaces were computed and compared with those of DOX and TC.^30^ The novelty of this work lies in exploring the influence of water on cycline adsorption. Two hypotheses were evaluated: (i) that water molecules inhibit antibiotic interaction with HA surfaces, and (ii) that antibiotics displace surface-bound water to interact directly with HA. Additionally, we evaluated interaction strength using the Quantum Theory of Atoms in Molecules (QTAIM), which enables investigation of interatomic interactions through the topological properties of electron density (ρ­(r)). This method provides a statistical interpretation of chemical bonding and nonbonding interactions, essential for elucidating adsorption mechanisms.^40^ The combination of DFT and QTAIM simulations with experimental data provides valuable insights into the role of water in cycline adsorption on HA surfaces. These findings contribute to the development of more effective and safer HA-based therapeutic systems.

### Materials and Methods

Synthesis, characterization of HA and mino (Sigma-Aldrich, Corp. Brazil) adsorption was conducted with the same methodology described in our previous article for dox and tc^30^. The time required to reach saturation was 6 h (Figure S1). Mino was downloaded free of charge from the protein databank using the code MIY (https://www.rcsb.org/ligand/MIY). Mino is composed by 4 rings (Figure S2) and present two UV–visible bands.^41,42^

### Computational Details

Theoretical studies were performed using SIESTA code^43^ by solving the kohn-sham equations^44^ with Troullier-Martins norm-conserving pseudopotentials,^45^ the PBE functional,^46,47^ the double ζ (DZP with polarization) basis set type with a 250 Ry for the cutoff energy and using 1 × 1x1 k-points. The force tolerance was set to 0.01 1/eV. The HA surfaces were modeled as slabs with 60 Å of vacuum perpendicular to the surface and infinitely repeated laterally considering periodical boundary conditions. The vacuum distance considered was enough to avoid interactions between specular images including a slab dipole correction.^48^ The interactions between the molecule and surfaces were evaluated using CRITIC2 ^49–52^ software to describe the interaction strength in terms of the electron density and its derivates using quantum theory of atoms in molecules (QTAIM)^53–58^ using the charge density computed by siesta. QTAIM approach offers valuable insights in the identification of the interactions from a theoretical perspective in terms of the electronic density. Quantification of intra- and intermolecular interactions is one of the most widely used tools for analyzing chemical interactions, offering valuable insights into the nature of these interactions.

## Results and Discussion

### Characterization of HA and Mino Adsorption

Mino adsorption isotherm was fitted using the Langmuir–Freundlich Kinect model of adsorption (Figure 1) given by eq 1):

$$a=\frac{a_{m}{KC}_{e}^{r}}{1+{KC}_{e}^{r}}$$ 1

where *a* is the equilibrium concentration, *K* is the equilibrium constant, a_m_ is the maximum amount of drug adsorbed, C_e_ is the concentration on the solution and *r* is a cooperative factor that express the molecule–molecule binding energy interaction^59^ (r = 1 for noninteracting, *r* > 1 a positive cooperativity and 0 < *r* < 1 a negative cooperativity). The maximum concentration of adsorbed mino was 16.31 ± 0.28 mg mino/g HA, with the equilibrium constant being 3.53 ± 0.57. Furthermore, the results demonstrate positive cooperativity (r = 1.31 ± 0.23). The cooperative values were close to unity, indicating the occurrence of physicochemical monolayer adsorption without aggregation of molecules^60^

**Figure 1:**
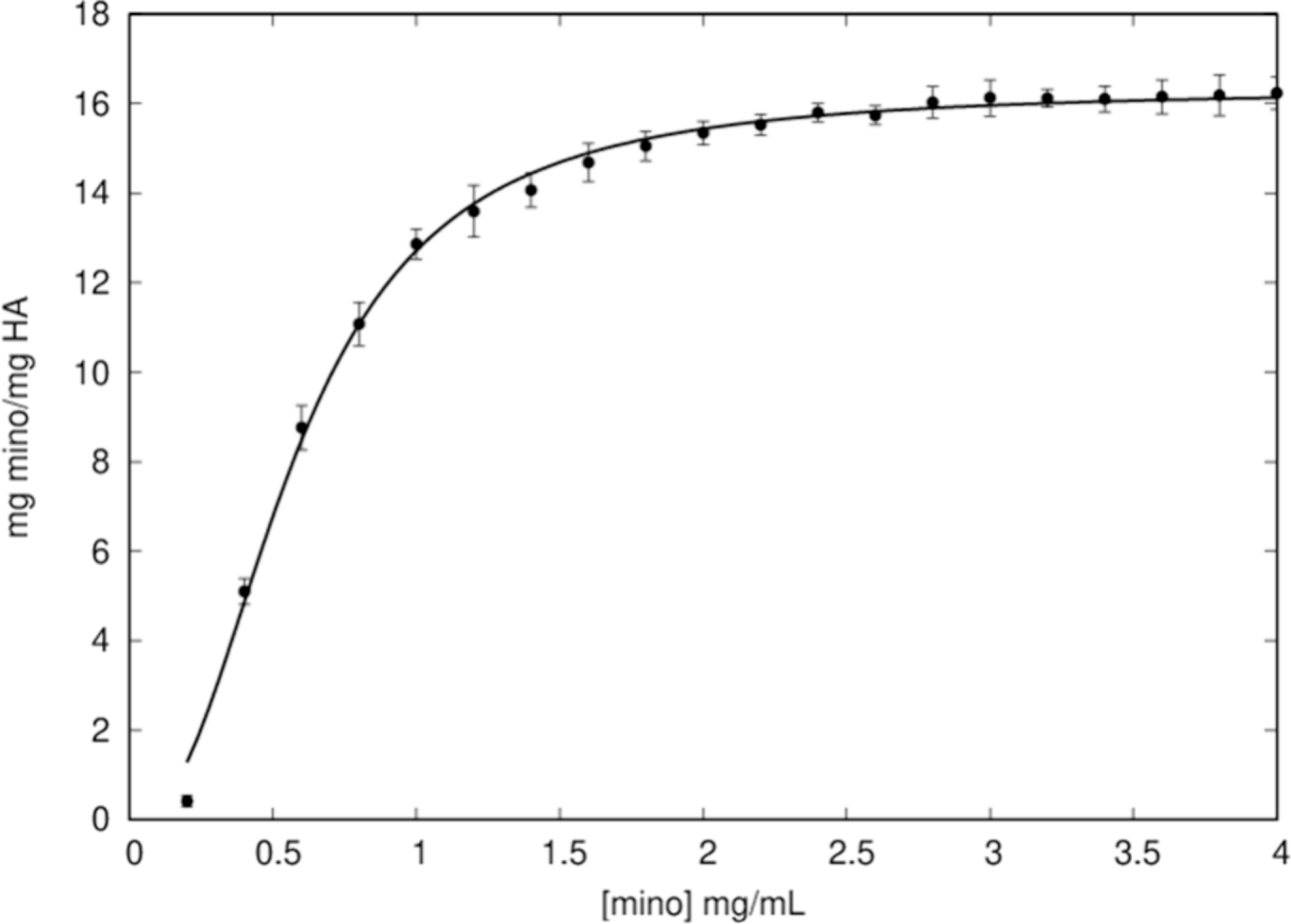
Adsorption isotherm curve for mino.

In a previous adsorption study using the same HA sample and experimental methodology,^30^ we showed that for dox, the maximum level of adsorption was 14.59 ± 0.57, whereas for tc, it was 9.62 ± 1.76. The cooperative values were close to unity, which also applies to minocycline. These findings indicate that the process occurs via physicochemical monolayer adsorption, with the molecules not aggregating. A comparison of the maximum concentrations shows that mino uptake is more significant than dox and tc. In terms of adsorption time, it took 4 h for tc to saturate the HA surface, compared to 6 h for both dox and minocycline.

### Atomistic Simulations

#### Mino Structure and Electrostatic Potential

minocycline is composed of a resonant structure resulting from the conjugation between C=O groups (C1=O1, C3=O2) and OH groups (O3), interconnected by a C=C bond (C2–C4). Furthermore, another resonant structure is evident in the α,β-unsaturated system of O5–C10=C11–C14=O6 (Figure S3). The molecular conformation was validated through a detailed examination of bond lengths and bond angles, which revealed equivalent bond lengths within the expected ranges and comparable values to those observed in related structures.^30,40,61–64^

Additionally, the bond distances between the relevant atoms were found tobe 1.27 Å (C1=O1), 1.24 Å (C3=O2), and 1.34 Å (C10–O5). Furthermore, the distance between the C4 and O3 atoms was determined to be 1.30 Å. The bond lengths in the α,β-unsaturated system were as follows: C=O (O5=C10), 1.34 Å; C=C (C10–C11), 1.37 Å; C–C (C11–C14), 1.47 Å; and C–OH (C14–O6), 1.26 Å. X-ray and neutron diffraction studies indicated that the bond distances of O=C, C–C, C = C, and C–O were 1.22, 1.46, 1.36, and 1.33 Å, respectively, for the C=C–OH systems,^65^ suggesting that the tautomeric form with a C=O group at O30=C29, a C=C at C11–C14, and an OH group at O6 is not present.

A study of the electrostatic potential^66^ was conducted in order to evaluate the potential adsorption regions of the molecules. This method is especially beneficial for determining the reactive areas of a molecular structure and offers a valuable approach to visualizing relative polarities. Furthermore, the electrostatic potential V­(r) is well-suited for examining processes involving the recognition of one molecule by another, as is the case with drug receptors. This was plotted based on a constant-electron-density surface mapped using an electrostatic-potential contour map in accordance with standard procedure. The varying values of the electrostatic potential are depicted using a color-coded representation, with red indicating stronger repulsion, green indicating intermediate levels and blue indicating stronger attraction.

In other words, the negative regions relate to nucleophilic reactivity, with the electron donor shown in red, and the positive ones to electrophilic reactivity, with the electron acceptor shown in blue. In the case of minocycline (Figure 2), the nucleophilic regions are located on the oxygen atoms of the OH and C=O groups. By contrast, the electrophilic sites are situated on the Hydrogens of the primary amine (NH_2_) and hydroxyl (OH) groups, indicated by the blue region on the diagram.

**Figure 2:**
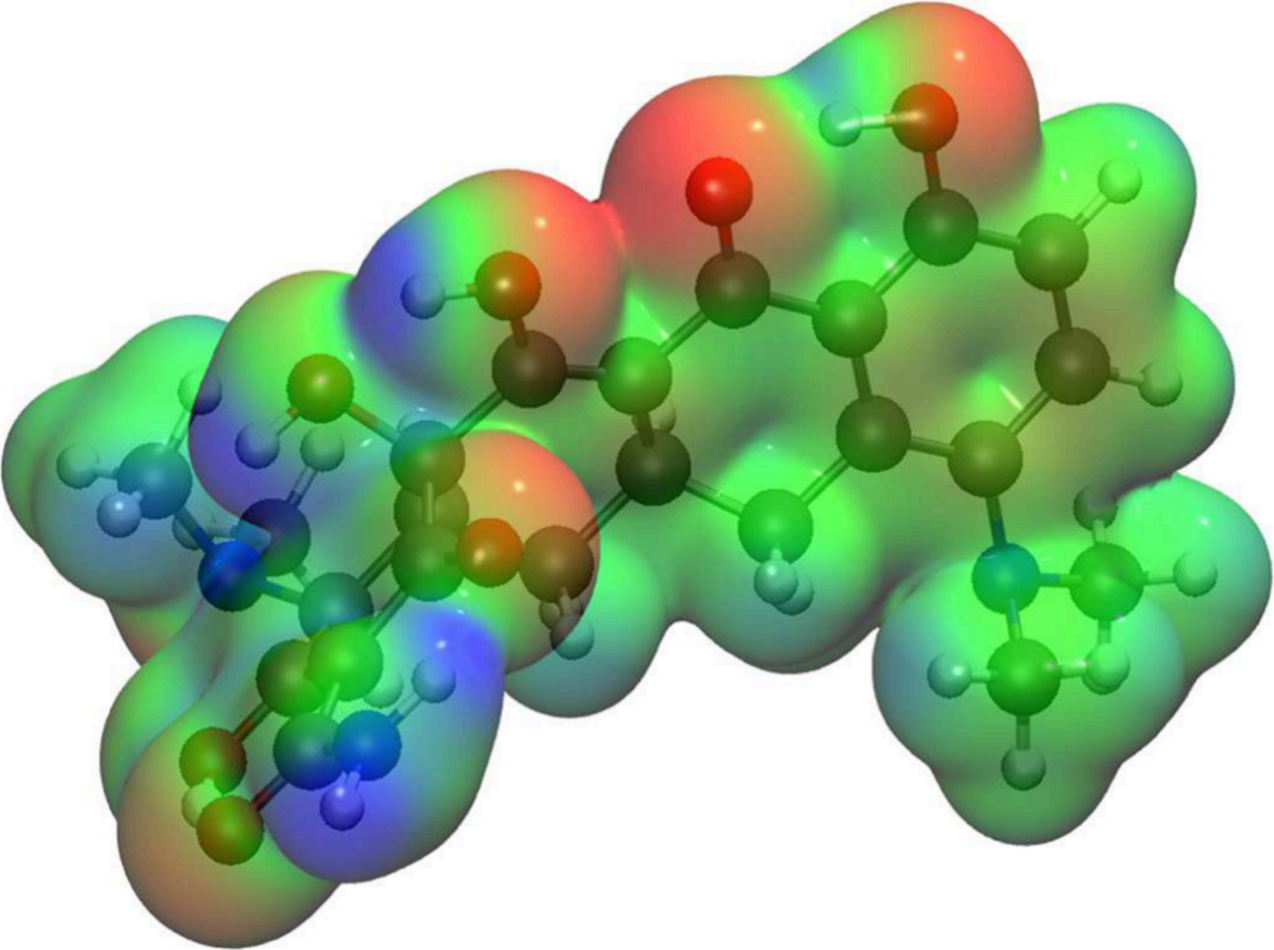
Molecular electrostatic potential of mino, where the red color is the nucleophilic region and blue is the electrophilic region visualized with VMD^67^

#### Adsorption Sites of Minocycline on a Dry HA Surface

The crystal structure of HA has a P6_3_/m space group, with the 4e Wyckoff positions occupied by two O atoms of the OH group, each with half occupancy. All slabs used for surface calculations are constructed on the basis of the optimized HA unit cell geometry. This study concentrated on two of the most crucial surfaces of apatite bone minerals: the (001) and (010) surfaces. These surfaces are significant in terms of mineral morphology, and they act as binding sites for a wide range of ionic species and small molecules. The slabs were modeled in accordance with the experimentally obtained (001) and (010) surfaces. In the case of the (010) surface, two different terminations were considered in accordance with the HRTEM results obtained by Ospina et al.^31^

In a previous study,^30^ we evaluated the reactivity of three distinct surfaces, designated as 001, PO_4_, and OH. The PO_4_ surface contains calcium atoms and PO_4_ groups, which we have classified as a PO_4_ rich surface. By contrast, the OH surface comprises Ca atoms, PO_4_ groups and OH groups, which has been defined as a PO_4_-deficient surface. Other theoretical studies have demonstrated that the (001) surface is the most energetically stable.^68^ The (001) surface was constructed using a 3 × 3 × 1 unit cell, translated along the xy plane. By contrast, the 010-OH and 010-PO_4_ surfaces were constructed using 3 × 1× 4 unit cells.

In all cases, a vacuum distance of 60 Å was included. Furthermore, the atomic positions of the slabs were optimized in accordance with standard procedure. The resulting geometries were then employed in a study of molecule adsorption onto various surfaces. Additionally, the electrostatic potentials of the various surfaces indicated that the nucleophilic region is the PO_4_ groups on the surface, while the electrophilic region is the OH and Ca atoms. This suggests that the OH and Ca atoms are electrophilic and could readily interact with the nucleophilic regions of mino during the adsorption process. Therefore, the O atoms and PO_4_ groups are nucleophilic and interact with the electrophilic regions of mino.

After careful consideration of the data presented and the electrostatic potential behavior outlined above with respect to mino, two different configuration options were identified for interaction with HA surfaces (Figure S4). The first configuration involves the minocycline molecule oriented in such a way that the β-hydroxyketone system on the HA surfaces is in direct contact. The second configuration is characterized by the amide and OH groups facing the HA surfaces. To obtain the lowest-energy surface/adsorbate configuration of the interaction between molecules and surfaces, the adsorption energy was calculated using

$$E_{ads}=E_{sys}-\left(E_{sup}+E_{mol}\right)$$ 2

where E_sys_ is the energy of the surface with adsorbed molecule, E_sup_ is the energy of the simulation cell containing only the surface, and E_mol_ is the self-energy of the molecules calculated in the same conditions in their optimized form. Thus, a negative adsorption energy indicates that the adsorption of the molecule at the surfaces is thermodynamically favorable. Table 1 shows the adsorption energy for all simulations conducted in this work.

**Table 1:** Adsorption Energy for Mino onto the Different Surfaces

mino (eV)	001	010-OH	010-PO_4_
1	–4.32	–4.22	–3.11
2	–3.37	–2.15	–2.12

The results clearly show that minocycline has a strong affinity for HA surfaces across a range of configurations and surfaces. Configuration 1 is the most stable, as evidenced by an adsorption energy of – 4.32 eV when applied to the 001-HA surface. Moreover, the same configuration on the 010-OH surface is 0.1 eV less energetic. It can be concluded that both of these configurations represent the optimal conditions for mino on HA surfaces. With regard to the interaction between mino and the surfaces, the drug interacts with the surfaces via calcium atoms and phosphate groups. Figure 3 illustrates the two most stable configurations. Figure 3-a illustrates configuration 1 on the 001 surface. The oxygen atoms of the carbonyl and alcohol groups interact with calcium atoms at distances ranging from 2.65 to 3.31 Å. Furthermore, an interaction between the nitrogen atom of the amide group and a calcium atom of the surface was observed, with a distance of 2.94 Å. It is noted that hydrogen bonds are formed between the OH groups and the PO_4_ groups of the surface, with a range of 1.59 to 1.98 Å.

**Figure 3:**
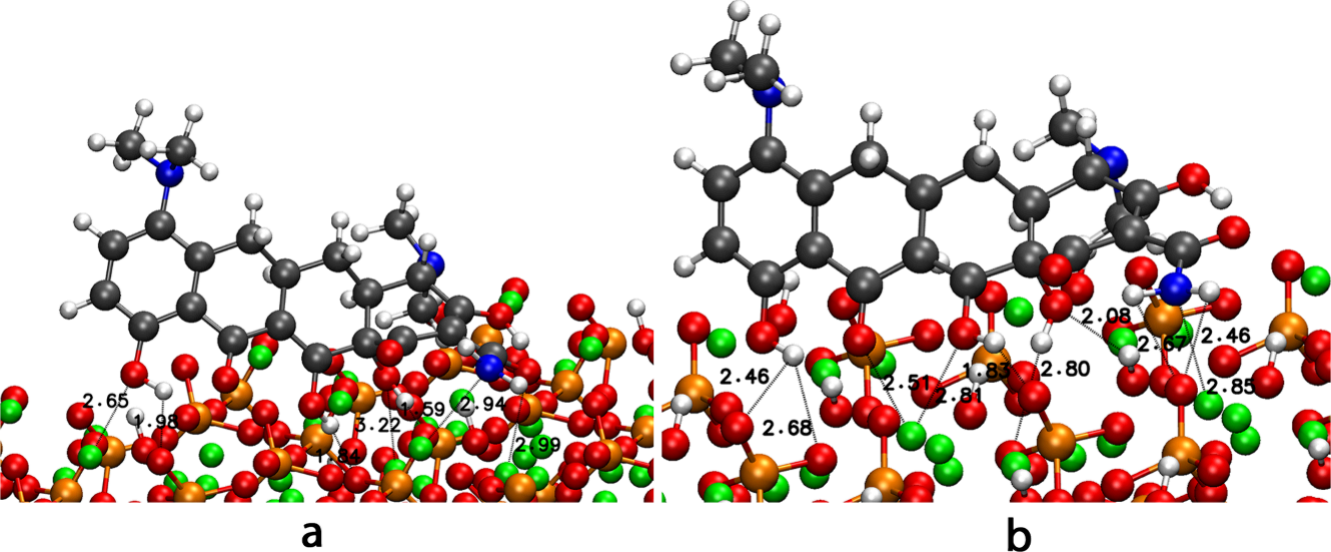
Most stable configurations of mino, configuration 1 in (a) 001-HA and (b) 010-OH HA surface.

In relation to the second configuration (Figure 3-b), the same adsorption sites are identified. The oxygen atoms of the molecule interact with the calcium atoms of the surface at a distance of between 2.51 and 2.81 Å. The nitrogen atom of the amide group interacts with a calcium atom on the surface at a distance of 2.85 Å. Furthermore, a hydrogen bond is formed between the OH and PO_4_ groups at a distance of 1.83–2.80 Å. Additionally, the hydroxyl groups on the surface interact with an OH group on the molecule at a distance of 2.08 Å. The PO_4_ groups on the surface interact with the hydrogen atoms of the amide group at distances of 2.67 and 2.46 Å.

Considering the other 4 configurations it is possible to affirm that the adsorption sites for HA surfaces are the calcium atoms interacting with oxygen atoms of the molecule and via hydrogen bonds with PO_4_ groups. Comparing these results with those obtained for dox and tc,^30^ it is possible to observe the same adsorption sites and distance range for the three different surfaces were obtained. However, mino shows interactions with hydroxyl groups of the surface in contrast with dox and tc that did not interact with them. In terms of adsorption energies for the nonhydrated systems reveals that mino has more configurations with better affinity than dox and tc. The most stable configuration is for mino on the 001 surface, meanwhile dox and tc prefer to interact with the PO_4_ surface. All configurations of mino onto the 001 and OH surfaces have more affinity than the dox and tc configurations. As described in the previous study, dox has a greater affinity for the HA surfaces than tc in terms of adsorption energies and also experimentally in terms of adsorbed concentration (14.59 ± 0.57 for dox and 9.62 ± 1.76 for tc). The experimental and the theoretical results, of both studies, follow the same behavior. mino is adsorbed in more quantity and also has more configurations with better affinity for HA surfaces than dox and tc. It confirms that mino is the cycline with more affinity for HA surfaces, followed by dox and tc is the cycline with the less affinity.

#### Water Adsorption on HA Surface

Hydroxyapatite has a high affinity for water molecules, and the antibiotics adsorption of antibiotics occurs on a hydrated HA surface. In addition, biological processes take place in a hydrated media at pH **≈** 7. In these conditions, it is mandatory to understand the adsorption of water molecules on the HA surface. Corno et al.^36^ conducted a study in which the water adsorption was carried out in stoichiometric (001) and (010) surfaces considering a water coverage range depending on the number of Calcium atoms on the different surfaces. They observed that the oxygen atoms of water molecules want to interact with calcium atoms, and Hydrogen atoms interact via hydrogen bonds with the Oxygen atom of the PO_4_ molecules of HA. Nevertheless, they observed that 010 surface water dissociates without an apparent energy barrier.

In our case, we are working with new experimental 010 surfaces (PO_4_ and OH surfaces). We considered a full coverage of calcium atoms on the surface by water molecules. In other words, the number of water molecules in the unit cell is the same as calcium atoms in the surface. In the case of the 001 surface, we included nine water molecules on the surface at a distance of 2.5 Å from the calcium atoms, and we optimized the system until we reached the convergence criteria. An interaction between Oxygen atoms of water molecules and Calcium atoms on the surface is observed. The distance of these interactions is 2.51 Å. A hydrogen bond between the hydrogen atom of water molecules and oxygen atoms of PO_4_ is also observed with a distance of 1.72–1.73 Å (Figure 4-a). PO_4_-010 surface contains 20 water molecules on top of calcium atoms of the surface, obtaining the same interactions as in the 001 surface with a distance of 2.59–2.62 Å for Ca–O and 1.66–1. Å for hydrogen bonds between PO_4_ molecules and hydrogen atoms of water molecules (Figure 4-b). OH-010 surface (Figure 4-c) also contains 20 water molecules on the surface with distances of 2.64 −2.66 Å and 1.94–2.1 Å for Ca···O and PO_4_···H interactions, respectively.

**Figure 4:**
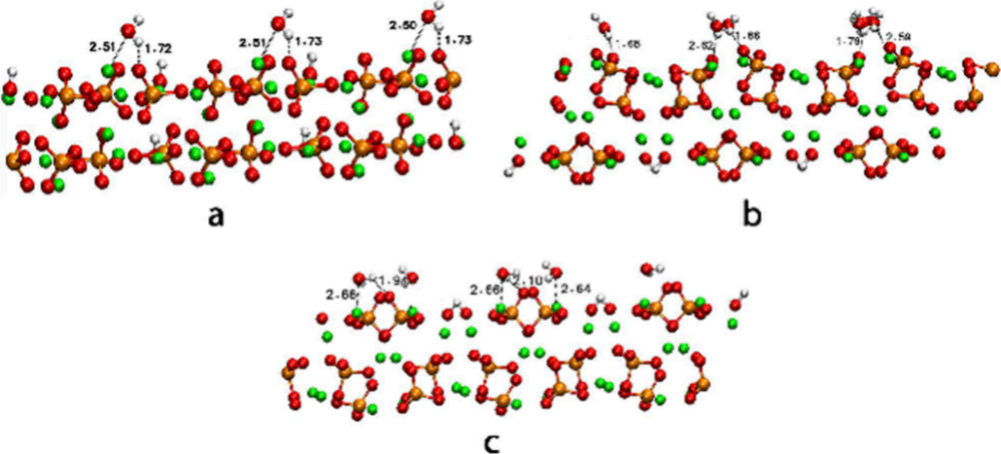
Water adsorption in (a) 001, (b) PO4, and (c) OH surfaces.

The main difference between these results and Corno’s is that we do not observe the dissociation of water molecules in any of the two 010 surfaces. One possibility for this discrepancy is the consideration of the long-range van der Waals correction in our simulations via Grimme functions. Adsorption energies (Table 2). were computed to reveal the affinity of water for the different surfaces using eq 2 but dividing by the number of water molecules in the system to compute the adsorption energy for a water molecule (*E*_ads_=[E_sys_-(E_sup_+E_wat_)]/n) where E_sys_ is the energy of the surface with adsorbed molecule, E_sup_ is the energy of the simulation cell containing only the surface, E_wat_ is the self-energy of a water molecule and n is the number of water molecules. Water molecules tend to adsorb strongly with the HA surfaces with adsorption energies from 0.81 up to – 0.91 eV. However, water molecules have more affinity for 001 than OH and PO_4_ surfaces.

**Table 2:** Adsorption Energy for Mino onto the Different Surfaces

surface	001	010-OH	010-PO_4_
water (eV)	–0.91	–0.87	–0.82

#### Water Effect on the Mino, Dox, and tc Adsorptions

This section describes the effect of water molecules on the noncovalent interactions of mino, dox, and tc with the hydrated hydroxyapatite (HA) surface. To this end, the optimized HA/water structures were modeled, including the different molecule conformations. The optimization parameters are the same as in the previous simulations carried out in this work. Table 3 reports the adsorption energies, showing the effect of water. Comparing the adsorption energies between the system with and without water, it is possible to observe that the water molecules do not inhibit the interaction between the molecules and the HA surfaces. It should be noted that a constraint has been taken into account in our approach. First, we optimize the HA/water system, and then this optimized system is brought into contact with the molecules. The constraint imposed is the complete coverage of the surface, which, in principle, prevents contact between molecules and HA surfaces.

**Table 3:** Adsorption energy for mino onto the different surfaces

	mino (eV)		dox (eV)			tc (eV)		
surface	1	2	1	2	3	1	2	3
001	–3.11	–2.85	–3.33	–3.35	–2.17	–4.07	–2.45	–2.60
010-OH	–2.35	–2.55	–2.28	–1.82	–1.81	–2.26	–2.07	–1.99
010-PO_4_	–3.18	–2.76	–2.90	–3.01	–2.84	–2.63	–1.79	–2.50

mino shows the most stable configuration for configuration 1 on surfaces 010 (Figure 5-a) and PO_4_ (Figure 5-b). The interactions correspond to the same interactions reported for the waterless system, except for the appearance of hydrogen bonds with the water molecules adsorbed on the surface. Ca···O interactions occur from 2.58 to 3.29 Å, and the OH group forms hydrogen bonds with the PO_4_ on the surface at 2.47 Å. Moreover, an interaction between OH and water molecules is observed with 1.87 Å. Comparing both systems, we find that the distance ranges are not appreciably different between the hydrated and nonhydrated systems. The difference in adsorption energy between the two most stable configurations is 0.07 eV, making the adsorption on the PO_4_ more stable. Considering the other configurations, it is possible to observe that some conformations are less stable in the hydrated system. Examples are configurations 1 and 2 on the 001 surface and configuration 1 on the OH-010 surface. This is because the water molecules adsorbed on the surface prevent these configurations from interacting in the most efficient way with the surface due to steric impediments. In contrast, water molecules stabilize some other configurations via hydrogen bonds.

**Figure 5:**
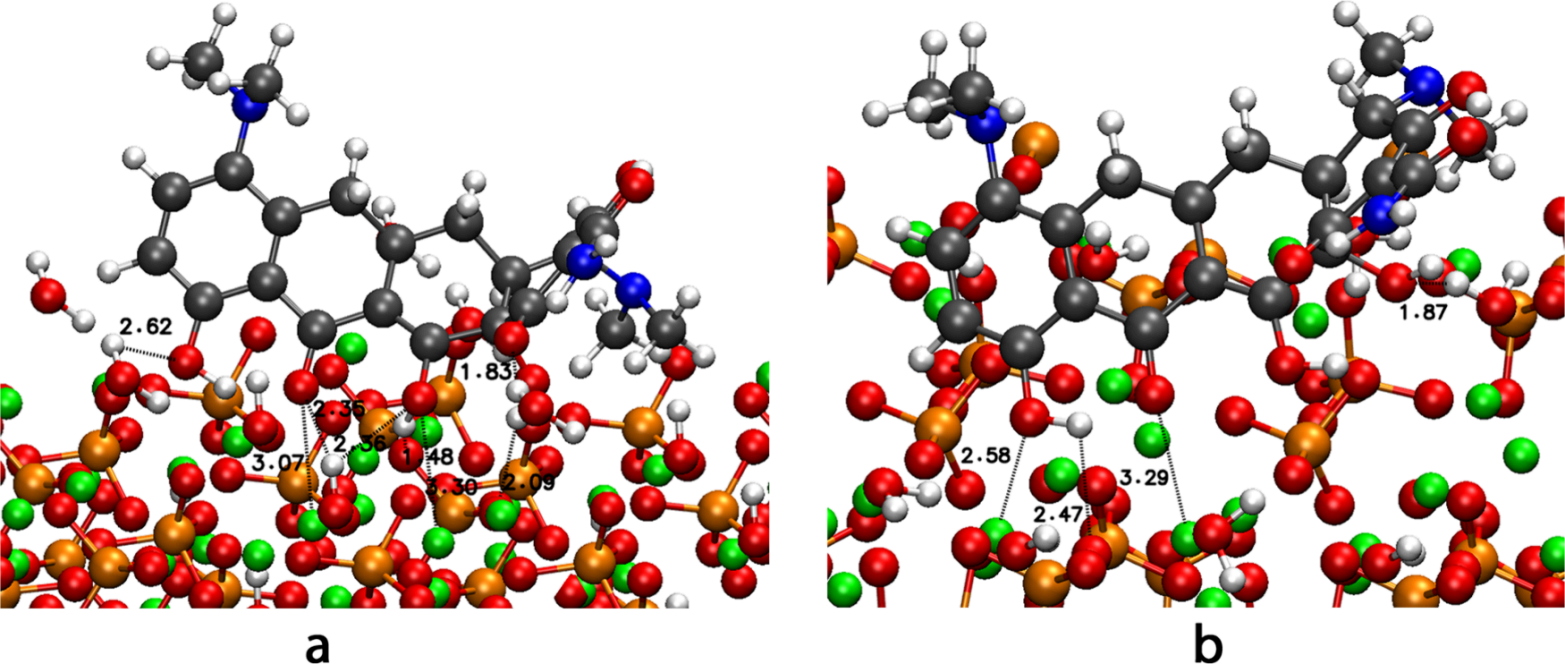
Most stable configurations of minocycline (a) configuration 1 on 001 and (b) configuration 1 on the PO_4_ HA-surface.

In the case of dox (Figure 6), the most stable configuration for the nonhydrated system is configuration 2 on the PO_4_-010 surface (−4.9 eV). However, the most stable configuration for the hydrated system is configurations 1 and 2 on the 001 surface. Configurations with poor affinity for the surfaces without water are stabilized by water molecules in hydrated HA surfaces. One possible explanation is the stabilization of dox through hydrogen bonds with water molecules. The distance ranges for Ca···O interactions vary from 2.46 to 3.10 Å and 1.44 to 2.15 for hydrogen bonds with PO_4_ molecules, which are very similar to the nonhydrated system and the ranges for mino. The formation of hydrogen bonds between dox and water molecules can be confirmed with distance intervals ranging from 1.76 to 2.29 Å. Configuration 1 on the 001 surface obtains a new interaction between N of the amide group and a calcium atom of the surface 3.31 Å, which was not found in the nonhydrated system.

**Figure 6:**
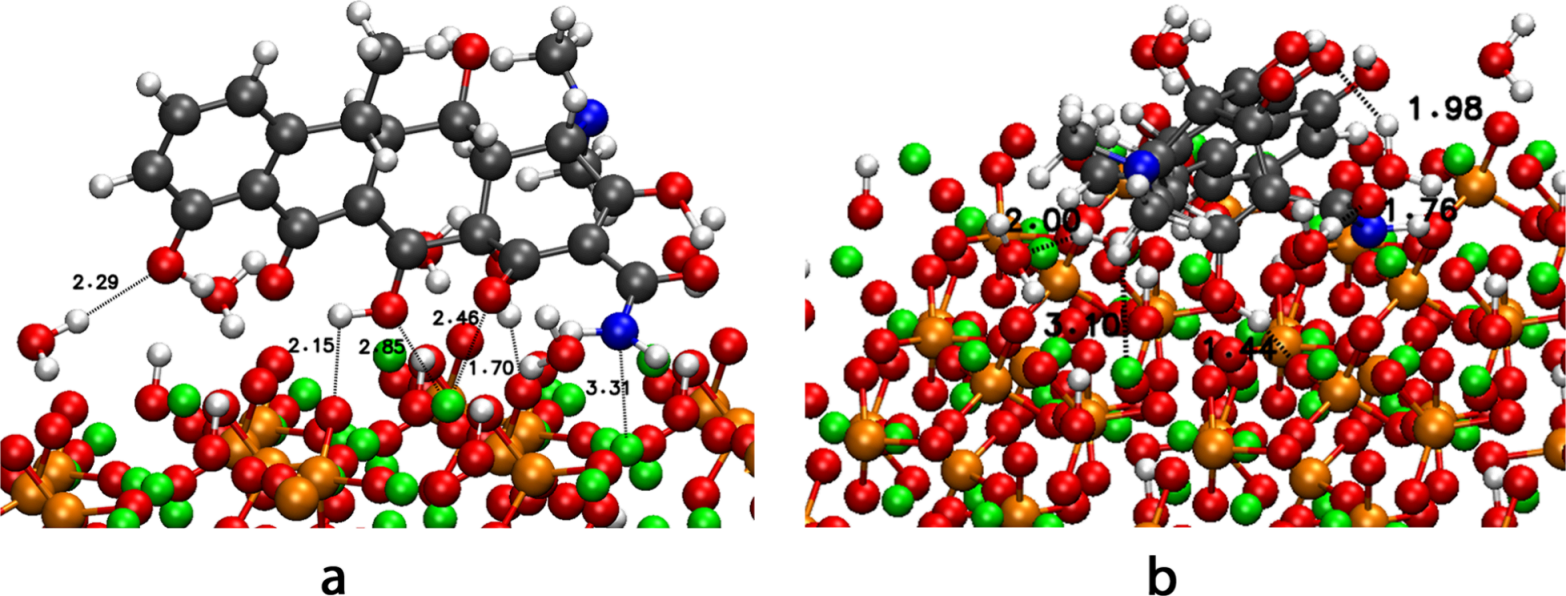
Most stable configurations of dox on 001 HA surface (a) configuration 1 and (b) configuration 2.

The tc shows more stable adsorption energies in the hydrated system than in the nonhydrated system due to the presence of water on the surface. The hydrogen bonds between the tc and the water molecules adsorbed on the surface stabilize the different configurations that were less stable in the nonhydrated system. The most stable configuration is configuration 1 on the 001 surface (Figure 7). As in the previous two cases, the distance ranges for the different interactions remain similar, with 2.46–3.29 Å for Ca–O interactions and 1.80–1.96 Å for hydrogen bonds with PO_4_ groups. This conformation does not present interactions between tc and water molecules.

**Figure 7:**
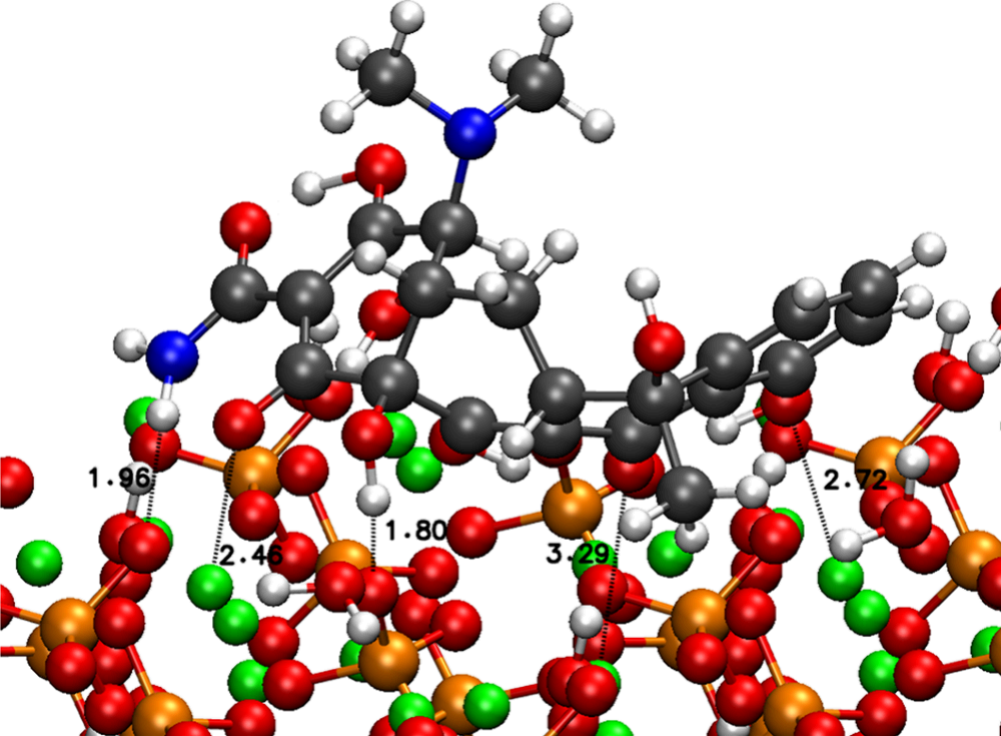
Most stable configurations of tc of configuration 1 on 001 HA.

Considering the information provided for the different conformations of the three molecules on the different surfaces, we can determine that a hydrated system does not avoid cyclins adsorption, stabilizing them through hydrogen bonds. In terms of adsorption sites, there are some destabilization and stabilization due to the presence of water molecules in the surface. As it has been shown, dox, tc, and mino have displaced water molecules from the different surfaces to interact with the HA. Therefore, we can affirm that the role of water molecules in the adsorption of the molecules is important and cannot be neglected in this work. The effect of the adsorbed water influences the preference of the antibiotics for the different surfaces. As shown previously, water molecules have a greater affinity for the 001 surfaces. This fact is shown in the affinity of the drugs for the hydrated 001 surface instead of PO_4_ (dox, tc and mino) or OH (mino) surfaces in a dry system. Also, some configurations with a poor affinity for the surfaces without water become more stable in the presence of water molecules.

#### Strength of the Noncovalent Interactions Using the QTAIM Approach

The noncovalent interaction strength was characterized by the quantum theory of atoms in molecules (QTAIM).^53–58^ It offers powerful information to understand the nature of hydrogen bonds; the central idea is the topological analysis of the electron density (ρ­(r)). Chemical bonding and nonbonding interactions between atoms result in an electron density deformation. QTAIM can map the interactions, allowing real-space partitioning into atomic regions called basins. This partitioning provides atomic properties, including atomic population and a statical interpretation of chemical bonds. The surface delimiting these basins is determined by electron density via the zero-flux condition (∇ρ­(r) n­(r)=0), where r is any point on the surface and n­(r) is the vector normal to it. The zero-flux condition ensures that atomic expectation values of momentum-based operators, such as kinetic energy obtained by integration over the basin, are uniquely defined. Critical points (CP’s) of the electron density, where (∇ρ­(r) n­(r)=0, hold particular significance. CP’s are categorized into nuclear critical points (NCP’s), where the maxima of the electron density is typically located at the nuclei, bond critical points (BCP’s), which correspond to the definition of a chemical bond (first-order saddle point), and second order saddle point of minima known as ring critical point (RCP’s) and cage critical point (CCP’s), respectively. The topology of the electron density, including the properties (density and Laplacian) at these critical points, has been linked to chemical concepts such as bond strength or covalent character.^54^ The central idea of this work is to analyze the BCPs resulting from the interactions between molecules and surfaces.

To this task, CRITIC2 ^49–52^ was designed for the topological analysis of the electron density in the solid state using the electron density calculated by SIESTA.^43^ Once the BCP’s are identified, their properties reveal important details about the nature of the interactions. Following Rozas’s criterion,^69^ the Laplacian of the electron density, ∇ρ­(r)^2^ ρ­(r), and the energy densities, G_bcp_ and V_bcp_, offer a window into classifying HB’s. In this system, different types of HB emerge, ranging from weak, electrostatically driven interactions (where ∇ρ­(r)^2^ ρ­(r) > 0 and H_bcp_ > 0), to medium-strength interactions (where ∇ρ­(r)^2^ ρ­(r) > 0 y H_bcp_ < 0) and even intense, covalent-like bonds where (both <0). Moreover, the -G/V ratio was used as criteria of HB descriptor^70,71^ where V and G are local electronic kinetic energy and the potential energy density per atomic unit volume at critical points, respectively. HB are not covalent for -G/V more than 1; between 0.5 and 1, it is partially covalent.^72^

We are interested in the noncovalent interactions between molecules and surfaces. First, we analyzed the dry systems. Afterward, we analyzed and compared the systems with water to determine the influence of the water molecules on the strength of the interactions between molecules and surfaces. Tables S1–S9 in the Supporting Information reveal the values of the different properties. Considering the systems without water molecules (dry systems), for mino 2 systems were selected, configuration 1 in 001 and OH surfaces. Figures S5–S13 and tables in Supporting Information, the topological graph are shown where only the BCP’s are displayed. In total 9 different configurations were considered in terms of adsorption energy, 4 configurations for the systems without water and 5 for the systems with water. For mino, the configuration 1 for the surfaces 001 and 010-OH were chosen due to they are the most stable when the system has no water. In the case that the water is in the media, the configuration 1 for the 001 and PO_4_ surfaces was considered. For dox, we have selected 3 different configurations. The configuration 2 in the PO_4_ surface for the system without water and the configurations 1 and 2 for the 001 surface when the system contains water. In the case of tc, only two configurations were considered, the configuration 1 in the PO_4_ surface for the nonhydrated system, and the configuration 1 onto the 001 surface for the hydrated system.

In general, all molecules present the same kind of interactions and they are in the range between electrostatic up to medium/strong strength interactions. For nonhydrated systems, the presence of the O···Ca, OH···O, O···O and CH···O interactions are found for the 4 configurations. Nevertheless, minocycline reveals another interaction that dox and tc does not present the NH···O interaction. Configuration 1 in the 001 surface for minocycline revels 13 BCP’s, interactions between calcium atom form the surface with oxygen atoms of the molecule with electrostatic character with a -G_bcp_/V_bcp_ ratio in the range of 1.1–1.16 (4 BCP’s). There is also, one critical point revealing the interaction between the calcium atom and a nitrogen of the molecule with an electrostatic character, but with a -G_bcp_/V_bcp_ less electrostatic than the Ca···O (1.09). With this result it is possible to justify that the interaction between calcium and nitrogen is stronger than calcium and oxygen. The OH···O interactions can be defined in two different types, from the molecule to the surface and viceversa. Considering the HB formed from the molecule to the surface (interaction 2 and 6 in Table S1 of Supporting Information) reveals an electrostatic behavior (-G_bcp_/V_bcp_ 1.05–1.21 and ∇ρ­(r)^2^ ρ­(r)> 0 and H_bcp_ > $0).

In counterparts, when the HB is formed from the surface to the molecule (interaction 8, 10 and 12), the nature of the interaction has a medium/strong strength (-G_bcp_/V_bcp_ 0.74–0.92, ∇ρ­(r)^2^ ρ­(r)> 0 H_bcp_< 0). Another HB is formed from the interaction between the NH group of the molecule and one oxygen atom of the phosphate groups of the surface (interaction 4). This interaction is less strong than the HB formed between OH groups of the molecule and the Oxygen atom of the surface (-G_bpc_/V_bpc_ 1.0 and ∇ρ­(r)^2^ ρ­(r) > 0 y H_bcp_ < 0). Another electrostatic interaction is found between the CH group of the molecule and the OH group of the surface (-G_bpc_/V_bpc_ 1.0 and ∇ρ­(r)^2^ ρ­(r)> 0 y H_bcp_ < 0).

In the case of configuration 1 of minocycline on the 010-OH surface 12 BCP’s are found (Figure S6, Table S2). Two of them are electrostatic interaction between calcium and oxygen of the molecule (-G_bpc_/V_bpc_ 1,11–1,16 and ∇ρ­(r)^2^ ρ­(r)> 0 and H_bcp_ > 0). The interaction between calcium and nitrogen atom of the molecule is found (-G_bpc_/V_bpc_ 1.11 and ∇ρ­(r)^2^ ρ­(r)> 0 and H_bcp_ > 0). The electrostatic interaction between CH group and oxygen atom of the surface is less energetic than in the previous configuration (-G_bpc_/V_bpc_ 1.12 and ∇ρ­(r)^2^ ρ­(r)> 0 and H_bcp_ > 0) but in this configuration an interaction between one methyl group of the molecule and a calcium atom is found with an electrostatic behavior (-G_bpc_/V_bpc_ 1.03 and ∇ρ­(r)^2^ ρ­(r) > 0 and H_bcp_ > 0) and also between a nitrogen atom and an oxygen atom of the surface (-G_bpc_/V_bpc_ 1.08,∇ρ­(r)^2^ ρ­(r)> 0, H_bcp_> 0). In this configuration, six OH ··· O interactions are found where two of them are from the molecule to the surface but in this case interaction 1 has an electrostatic behavior (-G_bpc_/V_bpc_ 1.13 and ∇ρ­(r)^2^ ρ­(r)> 0 and H_bcp_ > 0) and interaction 8 has medium strength (-G_bpc_/V_bpc_ 0.95 and ∇ρ­(r)^2^ ρ­(r)> 0 and H_bcp_ < 0). When the interaction is formed from the surface to the molecule a medium strength is obtained (-G_bpc_/V_bpc_ 0.85–0.96 and ∇ρ­(r)^2^ ρ­(r)> 0 and H_bcp_ < 0).

The most stable configuration of dox (Figure S7, Table S3) present 18 CP’s with 9 electrostatic interactions as CH···O (-G_bpc_/V_bpc_ 1.0–1.08 and ∇ρ­(r)^2^ρ­(r) > 0 and H_bcp_> 0), 2 O···O electrostatic interactions (-G_bpc_/V_bpc_ 1.0–1.24 and ∇ρ­(r)^2^ρ­(r)> 0 and H_bcp_ > 0), 3 Ca···O electrostatic interactions (-G_bpc_/V_bpc_ 1.04–1.15 and ∇ρ­(r)^2^ρ­(r) > 0 and H_bcp_ > 0). Two Ca····O electrostatic interactions (-G_bpc_/V_bpc_ 1.02–1.13 and ∇ρ­(r)^2^ ρ­(r) > 0 and H_bcp_ > 0). Two medium strength interactions between CH groups of the molecule and calcium atoms of the surface (-G_bpc_/V_bpc_ 0.96–0.99 and ∇ρ­(r)^2^ ρ­(r)> 0 and H_bcp_ < 0) and the strongest interaction is the NH···O (-G_bpc_/V_bpc_ 0.80 and ∇ρ­(r)^2^ ρ­(r)> 0 and H_bcp_ < 0). With this information it is possible to affirm that the most stable configuration of dox is regulated by electrostatic interactions between the molecule and the surface in contrast with the two most stable positions of dox that the medium/strong strength interactions are predominant. This feature is corroborated not only by the adsorption energies but also by the experimental results in the amount adsorbed the cyclines on the HA surfaces.

In the case of tc (Figure S8, Table S4), 11 BCP’s are obtained. It presents 4 electrostatic O···O interactions (-G_bpc_/V_bpc_ 1.09–1.25 and ∇ρ­(r)^2^ ρ­(r)> 0 and H_bcp_ > 0), 3 Ca···O interactions (-G_bpc_/V_bpc_ 1.12–1.16 and ∇ρ­(r)^2^ ρ­(r)> 0 and H_bcp_ > 0), two CH···O electrostatic interactions (-G_bpc_/V_bpc_ 1.13–1.17 and ∇ρ­(r)^2^ ρ­(r)> 0 and H_bcp_ > 0), two medium/strong strength interactions between the NH···O (-G_bpc_/V_bpc_ 0.77 and ∇ρ­(r)^2^ ρ­(r)> 0 and H_bcp_ < 0) and OH···O (-G_bpc_/V_bpc_ 0.62 and ∇ρ­(r)^2^ ρ­(r) > 0 and H_bcp_ < 0). The OH···O interaction is a HB formed from the surface to the molecule and the strongest interaction obtained for the most stable configuration of tc. As in the case of dox, tc is stabilized by electrostatic interactions and it has a smaller number of that interactions than dox. This effect also is reflected in the quantity of tc adsorbed experimentally. These results are in good agreement between experiment and adsorption energies.

Considering the hydrated systems, two configurations were selected for mino and dox and one for tc based on the adsorption energies. First, we have focused in the description of the interactions between molecules and surface. First configuration to describe is the configuration 1 on the 001 surface for minocycline. This configuration is described by 11 BCP’s between molecule and surface (Figure S9 and Table S5 in Supporting Information). This configuration has 6 OH···O interactions, where 3 are from the molecule to the surface (7, 8 and 9) and other 3 from the surface to the molecule (4, 6 and 10). All HB from the surface to the molecule are medium/strong strength (-G_bpc_/V_bpc_ 0.69–0,92 and ∇ρ­(r)^2^ ρ­(r)> 0 and H_bcp_ < 0) meanwhile the HB from the molecule to the surface are also medium/strong strength character (-G_bpc_/V_bpc_ 0.86–0,97 and ∇ρ­(r)^2^ ρ­(r)> 0 and H_bcp_ < 0). Again, HB from the surface to the molecule are stronger than the HB from the molecule to the surface. Also a CH3···Ca electrostatic interaction was found (-G_bpc_/V_bpc_ 1.06 and ∇ρ­(r)^2^ ρ­(r) > 0 and H_bcp_ > 0), two CH3···O electrostatic interactions (-G_bpc_/V_bpc_ 1.08–1.12 and ∇ρ­(r)^2^ ρ­(r) > 0 and H_bcp_ > 0) and one CH3···HO electrostatic interaction (-G_bpc_/V_bpc_ 1.0 and ∇ρ­(r)^2^ ρ­(r) > 0 and H_bcp_ > 0). The second configuration for mino is the configuration 1 on the PO4 surface (Figure S10 and Table 6 Supporting Information). Six CP’s are found, which all of them has electrostatic behavior. Three of them are the O···O interactions (-G_bpc_/V_bpc_ 1.06–1.38 and ∇ρ­(r)^2^ ρ­(r) > 0 and H_bcp_ > 0). Two O···Ca interactions (-G_bpc_/Vbpc 1.07–1.1 and ∇ρ­(r)^2^ ρ­(r) > 0 and H_bcp_ > 0) and one CH···O interaction (-G_bpc_/V_bpc_ 1.08 and ∇ρ­(r)^2^ ρ­(r)> 0 and H_bcp_ > 0).

The most stable configuration of dox, configuration 2 on the 001 surface can be described with 11 BCP’s (Figure S11 and Table S7 in Supporting Information). This configuration is composed by two NH···O interaction (-G_bpc_/V_bpc_ 1.06–1.09 and ∇ρ­(r)^2^ ρ­(r)> 0 and H_bcp_ > 0) and two OH···O interactions with medium/strong strength character (-G_bpc_/V_bpc_ 0,79–0,80 and ∇ρ­(r)^2^ ρ­(r)> 0 and H_bcp_ < 0), In counterparts three critical points has electrostatic character (-G_bpc_/V_bpc_ 1,01–1,14 and ∇ρ­(r)^2^ ρ­(r) > 0 and H_bcp_ > 0) and correspond with the O···Ca interactions. There is an electrostatic interaction (-G_bpc_/V_bpc_ 1,4 and ∇ρ­(r)^2^ρ­(r)> 0 and H_bcp_ > 0) that correspond with the interaction OH···Ca. Also, N···Ca interaction is found (-G_bpc_/V_bpc_ 1,0 and ∇ρ­(r)^2^ ρ­(r)> 0 and H_bcp_ < 0) with medium strength. The last two interactions have electrostatic character and correspond with the interactions N···O and O···O.

The second configuration of dox on surface 001 reveals 17 BCP’s­(Figure S12 and Table S8 in Supporting Information). Seven of them are the CH···O interactions which 6 of the are electrostatic interaction (-G_bpc_/V_bpc_ 1,04–1,29 and ∇ρ­(r)^2^ ρ­(r) > 0 and H_bcp_ > 0) and one of them have medium strength (interaction 7 -G_bpc_/V_bpc_ 0,99 and ∇ρ­(r)^2^ ρ­(r)> 0 and H_bcp_ < 0). Two CH···OH interactions with medium strength character (-G_bpc_/V_bpc_ 0,85–0,90 and ∇ρ­(r)^2^ ρ­(r)> 0 and H_bcp_ < 0). Two O···Ca interactions (-G_bpc_/V_bpc_ 1,07–1,13 and ∇ρ­(r)^2^ ρ­(r)> 0 and H_bcp_ > 0), one N···Ca interaction (-G_bpc_/V_bpc_ 1,07 and ∇ρ­(r)^2^ ρ­(r)> 0 and H_bcp_ > 0), one CH···Ca (-G_bpc_/V_bpc_ 0,99 and ∇ρ­(r)^2^ ρ­(r)> 0 and H_bcp_ < 0), two O···O interactions (-G_bpc_/V_bpc_ 1,26–1,30 and ∇ρ­(r)^2^ ρ­(r)> 0 and H_bcp_ > 0) and N···O interaction (-G_bpc_/V_bpc_ 1,09 and ∇ρ­(r)^2^ ρ­(r)> 0 and H_bcp_ > 0). Also, a HB for OH···O interaction from the surface to the molecule is found (-G_bpc_/V_bpc_ 0,91 and ∇ρ­(r)^2^ ρ­(r)> 0 and H_bcp_ < 0).

The last configuration is the configuration 1 on the 001 surface for tc with 12 critical points (Figure S13 and Table S9 in Supporting Information). Only three interactions have medium/strong strength, CH···OH (-G_bpc_/V_bpc_ 0,89 and ∇ρ­(r)^2^ ρ­(r)> 0 and H_bcp_ < 0), OH···O (-G_bpc_/V_bpc_ 0,82 and ∇ρ­(r)^2^ ρ­(r)> 0 and H_bcp_ < 0) and the strongest interaction NH···O (-G_bpc_/V_bpc_ 0,5 and ∇ρ­(r)^2^ ρ­(r)> 0 and H_bcp_ < 0). With electrostatic character two O···O (-G_bpc_/V_bpc_ 1,09–1,19 and ∇ρ­(r)^2^ ρ­(r)> 0 and H_bcp_ > 0), three O···Ca (-G_bpc_/V_bpc_ 1,15–1,19 and ∇ρ­(r)^2^ ρ­(r)> 0 and H_bcp_ > 0), two CH···O (-G_bpc_/V_bpc_ 1,08–121 and ∇ρ­(r)^2^ ρ­(r)> 0 and H_bcp_ > 0), on N···HO (-G_bpc_/V_bpc_ 1,06 and ∇ρ­(r)^2^ ρ­(r)> 0 and H_bcp_ > 0) and one C···O (-G_bpc_/V_bpc_ 1,22 and ∇ρ­(r)^2^ ρ­(r)> 0 and H_bcp_ > 0) have been obtained.

With all this information, for dry systems, minocycline showed strong and diverse interactions, including unique NH···O bonds not observed in doxycycline or tetracycline. The most stable configurations for minocycline were on the 001 and 010-OH surfaces, characterized by electrostatic Ca···O and Ca···N interactions, medium/strong H-bonds (OH···O), and weaker CH···O interactions. Doxycycline presented the most interactions in its stable configuration, with 18 BCPs dominated by electrostatic CH···O and O···O bonds, and medium/strong NH···O interactions. Tetracycline exhibited fewer interactions overall, with its stability governed by electrostatic O···O and Ca···O interactions and medium/strong NH···O and OH···O bonds. In hydrated systems, minocycline and doxycycline, maintained stability through multiple OH···O and O···Ca interactions, while tetracycline showed fewer but stronger interactions like NH···O and OH···O. The results highlight the predominance of electrostatic interactions in regulating adsorption and reflect experimental trends, with minocycline and doxycycline forming more complex and diverse interaction networks compared to tetracycline.

## Conclusions

In this study, we investigated the noncovalent interactions between the antibiotics minocycline (mino), doxycycline (dox), and tetracycline (tc) with hydroxyapatite (HA) surfaces through a computational approach utilizing the Quantum Theory of Atoms in Molecules (QTAIM) methodology. These interactions were analyzed in both dry and hydrated environments, complemented by experimental results and adsorption energy calculations. Our computational findings reveal that minocycline interacts most strongly with the HA surface, exhibiting a combination of electrostatic and hydrogen bonding interactions, including NH···O and OH···O bonds. These interactions are further supported by strong electrostatic interactions between calcium ions on the surface and the oxygen atoms of the molecules. The adsorption energy calculations indicate that minocycline shows the highest adsorption energy, confirming the dominance of these interactions in stabilizing the molecule on the HA surface. Doxycycline and tetracycline, although also demonstrating significant interactions, exhibit weaker and fewer hydrogen bonds, with tetracycline showing the lowest adsorption energies. This trend correlates with the experimental adsorption results, which show a higher adsorption of minocycline on the surface, consistent with the computational results.

The impact of hydration was also analyzed, showing that the presence of water molecules alters the hydrogen bonding network, enhancing the overall strength of surface-to-molecule interactions, particularly for minocycline. The experimental results support this, as hydration in aqueous solutions leads to stronger and more stable binding of the molecules, especially mino, to the HA surface. Additionally, the experimental data on the adsorption of these molecules in aqueous media corroborate the computational results, highlighting the importance of both electrostatic interactions and hydrogen bonding in determining the adsorption behavior. Water molecules, by modulating the interaction strength, contribute to the stabilization of the drugs on the HA surface, particularly in the case of mino, which exhibits a significant increase in interaction strength when hydrated.

In conclusion, the combination of computational simulations and experimental data provides a thorough understanding of the noncovalent interactions governing the adsorption of these drugs onto HA surfaces. mino, due to its strong hydrogen bonding and electrostatic interactions, shows the highest affinity for the HA surface, followed by doxycycline, while tetracycline exhibits the weakest interaction. This insight is valuable for the design of targeted drug delivery systems, where surface interactions play a crucial role in controlling the release and retention of therapeutic agents in bone mineral environments.

## Supplementary Material



## Notes

UV–vis spectra of minocycline, molecular structure of minocycline, molecular sketch with labels, configurations selected, molecular graph of CPs, and QTAIM parameters of BCPs for the main configurations (PDF)
