# Supplementary material for: Influence of Water on the Adsorption Sites of Cycline Antibiotics onto Hydroxyapatite Surfaces
Source: Langmuir. 2025 Sep 29;41(40):27330–40. doi: 10.1021/acs.langmuir.5c03448 (PMC12530039; doi:10.1021/acs.langmuir.5c03448)
Supplement: Supplementary file 1 [file la5c03448_si_001.pdf]

Supporting Information.

Influence of water on the adsorption sites of cyclines antibiotics onto hydroxyapatite surfaces.

R. Soria-Martínez* and Alexandre Malta Rossi

Centro Brasileiro de Pesquisas Físicas, Rio de Janeiro, 22290-180, Brazil.

E-mail: rsoria@cbpf.br

Contents

List of Figures

S1 UV-Vis spectra of Minocycline. S4

S2 Molecular structure of minocycline. Carbon in gray, Oxygen in red, Nitrogen in blue and Hydrogen atoms in white. S4

S3 Molecular sketch of minocycline with atomic labels S5

S4 configuration selected S5

S5 Molecular graph including the BCP in magenta for configuration 1 in 001 surface for minocycline. S6

S6 molecular graph including the bcp in magenta for configuration 1 in OH surface for minocycline. S7

S7 molecular graph including the bcp in magenta for configuration 2 in PO_4 surface for DOX. S8

S8 molecular graph including the bcp in magenta for configuration 1 in PO_4 surface for TC. S10

S9 molecular graph including the bcp in magenta for configuration 1 in 001 surface for mino. S11

S10 molecular graph including the bcp in magenta for configuration 1 in PO_4 surface for mino. S12

S11 molecular graph including the bcp in magenta for configuration 2 in 001 surface for dox. S13

S12 molecular graph including the bcp in magenta for configuration 2 in 001 surface for TC. S14

S13 molecular graph including the bcp in magenta for configuration 1 in PO_4 surface for TC. S16

List of Tables

S1 QTAIM parameters of the BCP's for the interactions between configuration 1 of mino and 001-HA. S6

S2 qtaim parameters of the bcp's for the interactions between configuration 1 of mino and 001-OH. S7

S3 qtaim parameters of the bcp's for the interactions between configuration 2 of dox and PO_4-ha. S9

S4 qtaim parameters of the bcp's for the interactions between configuration 1 of tc and PO_4-ha. S10

S5 qtaim parameters of the bcp's for the interactions between configuration 1 of mino and 001-ha. S11

S6 qtaim parameters of the bcp's for the interactions between configuration 1 of mino and PO_4-ha. S12

S7 qtaim parameters of the bcp's for the interactions between configuration 2 of dox and PO_4-ha. S13

S8 qtaim parameters of the bcp's for the interactions between configuration 2 of TC and 001-ha. S15

S9 qtaim parameters of the bcp's for the interactions between configuration 1 of TC and PO_4-ha. S16

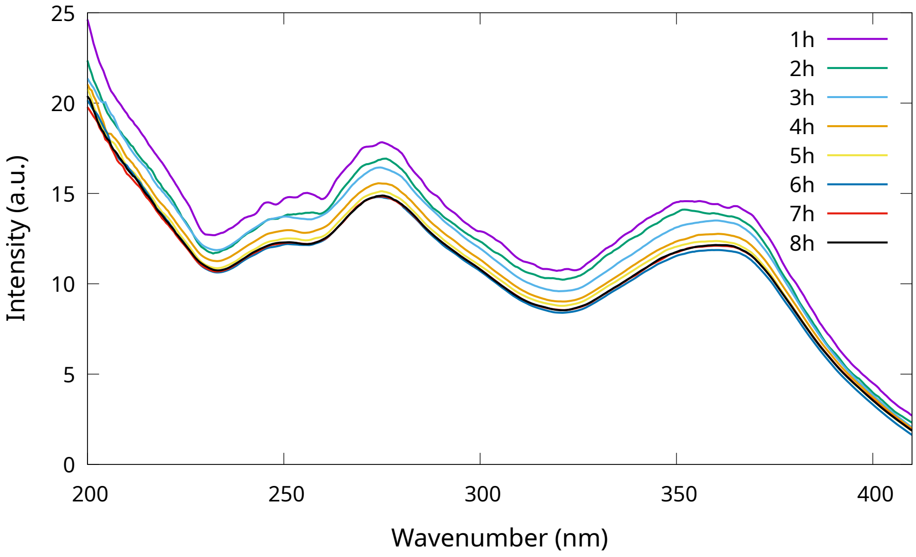

Figure S1: UV-Vis spectra of Minocycline.

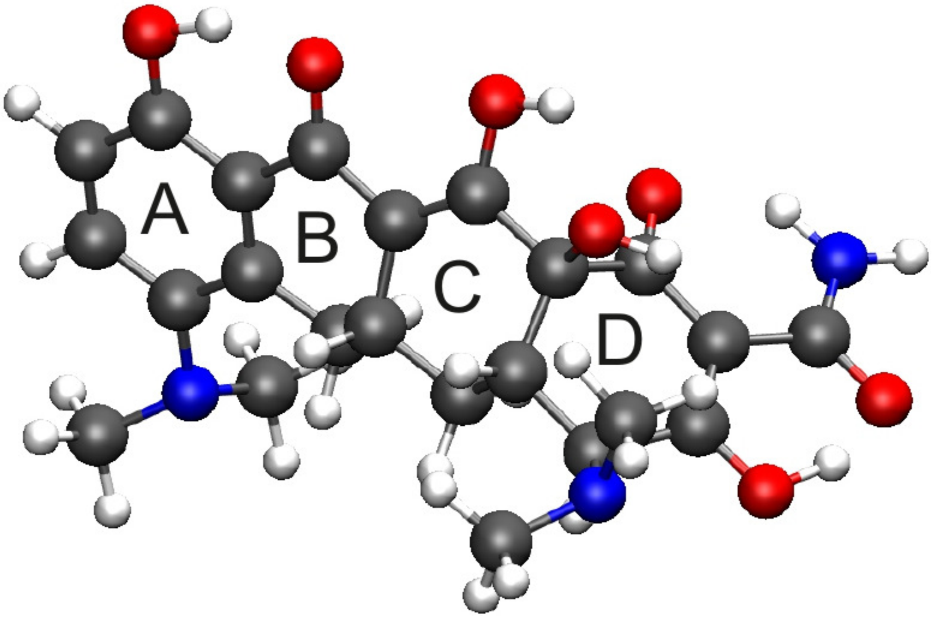

Figure S2: Molecular structure of minocycline. Carbon in gray, Oxygen in red, Nitrogen in blue and Hydrogen atoms in white.

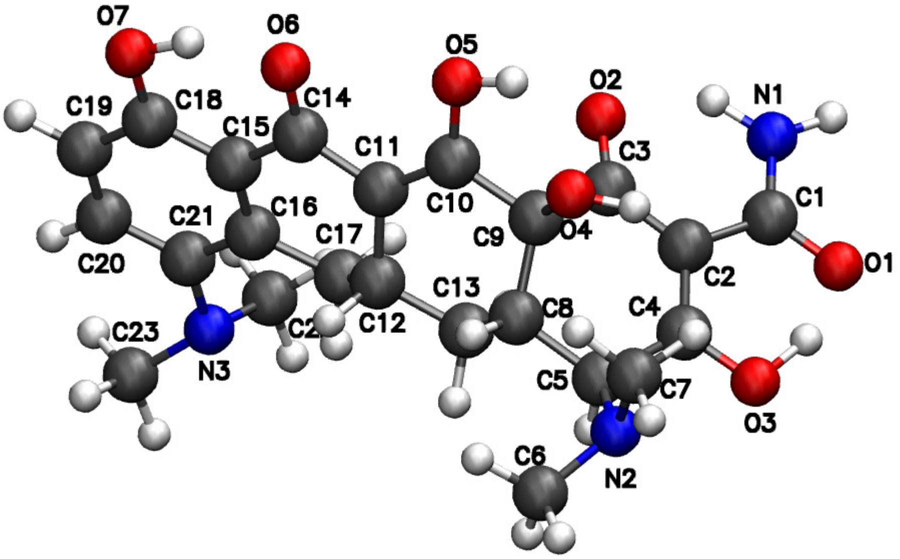

Figure S3: Molecular sketch of minocycline with atomic labels

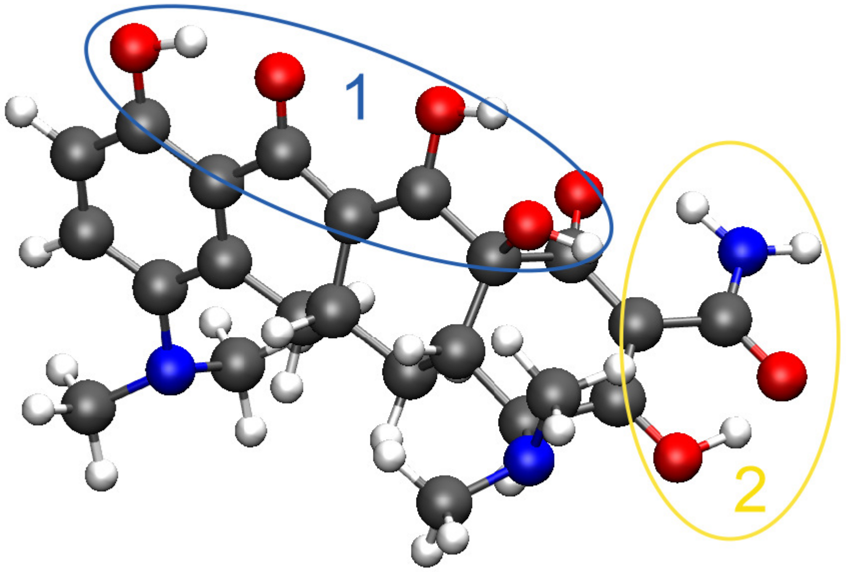

Figure S4: configuration selected

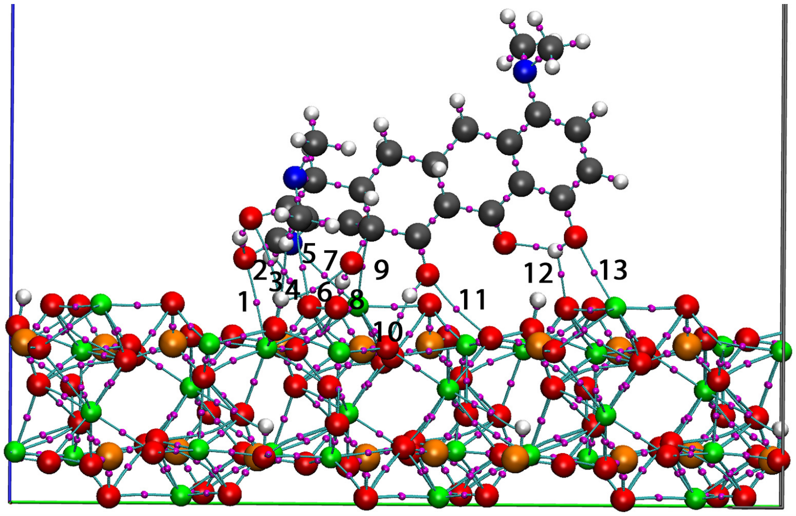

Figure S5: Molecular graph including the BCP in magenta for configuration 1 in 001 surface for minocycline.

Table S1: QTAIM parameters of the BCP's for the interactions between configuration 1 of mino and 001-HA.

Interaction	$X_{HA}\cdots X_{mino}$	ρ(r)	$\nabla^2\rho$(r)	G_{bcp}	V_{bcp}	H_{bcp}	$-G_{bcp}/V_{bcp}$
1	Ca$\cdots$O	0.0112	0.0267	0.0061	-0.0055	0.0006	1.1
2	O$\cdots$HO	0.0078	0.0191	0.0041	-0.0034	0.0007	1.21
3	CH$\cdots$HO	0.0115	0.0204	0.0051	-0.0051	0.0001	1.0
4	NH$\cdots$O	0.0161	0.0350	0.0088	-0.0088	-0.0001	1.0
5	CH$\cdots$O	0.0129	0.0378	0.0084	-0.0073	0.0011	1.15
6	OH$\cdots$O	0.0133	0.0303	0.0072	-0.0068	0.0004	1.05
7	Ca$\cdots$N	0.0138	0.0357	0.0082	-0.0075	0.0007	1.09
8	OH$\cdots$O	0.0682	0.1495	0.0576	-0.0779	-0.0202	0.74
9	Ca$\cdots$O	0.0128	0.0364	0.0081	-0.0071	0.0010	1.14
10	OH$\cdots$O	0.0419	0.1031	0.0317	-0.0376	-0.0059	0.84
11	O$\cdots$O	0.0114	0.0376	0.0079	-0.0065	0.0015	1.22
12	OH$\cdots$O	0.0321	0.0879	0.0239	-0.0260	-0.0021	0.92
13	Ca$\cdots$O	0.0187	0.0720	0.0158	-0.0134	0.0022	1.16

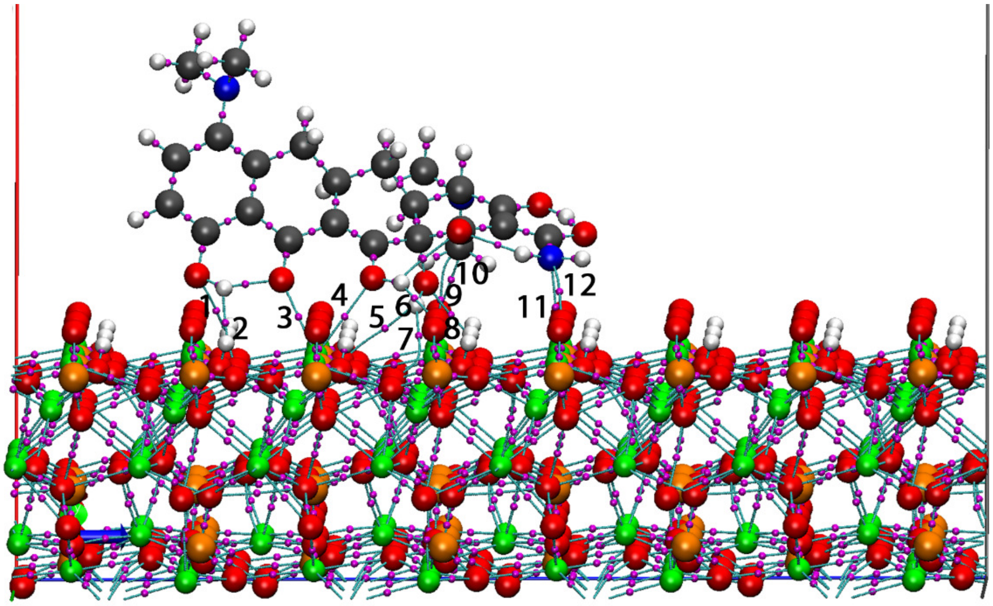

Figure S6: molecular graph including the bcp in magenta for configuration 1 in OH surface for minocycline.

Table S2: qtaim parameters of the bcp's for the interactions between configuration 1 of mino and 001-OH.

interaction	$x_{ha} \cdots x_{mino}$	ρ(r)	$\nabla^2\rho$(r)	g_{bcp}	v_{bcp}	h_{bcp}	$-g_{bcp}/v_{bcp}$
1	OH$\cdots$O	0.0114	0.0289	0.0065	-0.0057	0.0008	1.13
2	OH$\cdots$O	0.0150	0.0384	0.0090	-0.0094	-0.0007	0.95
3	O$\cdots$Ca	0.0237	0.1066	0.0234	-0.0201	0.0033	1.16
4	O$\cdots$Ca	0.0144	0.0455	0.0100	-0.0090	0.0013	1.11
5	OH$\cdots$O	0.0104	0.0215	0.0050	-0.0056	-0.0004	0.89
6	OH$\cdots$O	0.0413	0.1048	0.0317	-0.0371	-0.0055	0.85
7	OH$\cdots$O	0.0112	0.0211	0.0051	-0.0053	-0.0001	0.96
8	OH$\cdots$O	0.0269	0.0729	0.0191	-0.0199	-0.0009	0.95
9	$CH_3\cdots$Ca	0.0146	0.0331	0.0080	-0.0078	0.0003	1.03
10	CH$\cdots$O	0.0134	0.0336	0.0078	-0.0071	0.0006	1.12
11	Ca$\cdots$N	0.0155	0.0458	0.0104	-0.0093	0.0011	1.11
12	O$\cdots$N	0.0180	0.0559	0.0129	-0.0119	0.0010	1.08

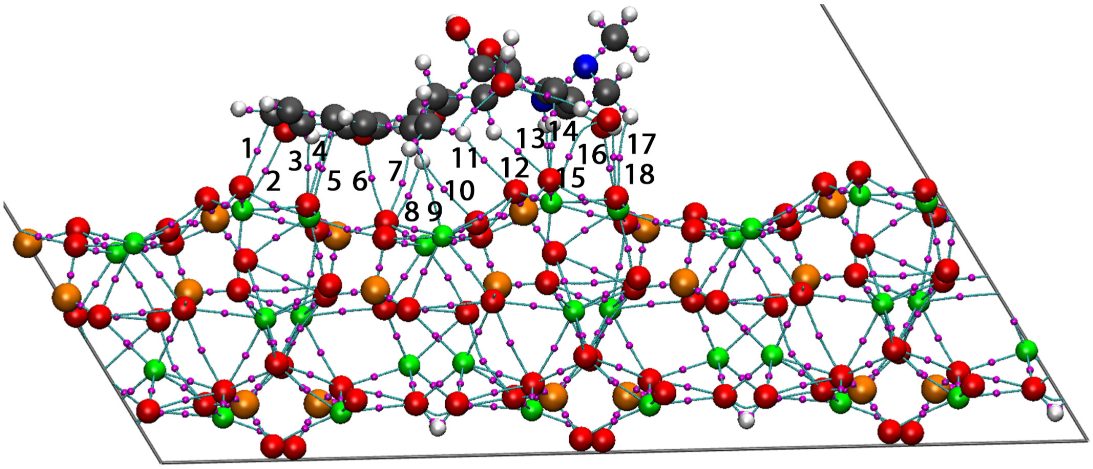

Figure S7: molecular graph including the bcp in magenta for configuration 2 in PO_4 surface for DOX.

Table S3: qtaim parameters of the bcp's for the interactions between configuration 2 of dox and PO_4-ha.

interaction	$x_{ha} \cdots x_{mino}$	ρ(r)	$\nabla^2\rho$(r)	g_{bcp}	v_{bcp}	h_{bcp}	$-g_{bcp}/v_{bcp}$
1	C···O	0.0259	0.0831	0.0203	-0.0199	0.0004	1.02
2	Ca···O	0.0143	0.0414	0.0097	-0.0085	0.0012	1.14
3	CH···O	0.0386	0.0961	0.0287	-0.0280	0.0046	1.02
4	C···O	0.0095	0.0220	0.0049	-0.0043	0.0001	1.13
5	CH···Ca	0.0113	0.0198	0.0049	-0.0050	-0.0001	0.99
6	O···O	0.0122	0.0440	0.0092	-0.0074	0.0018	1.24
7	CH···O	0.0111	0.0245	0.0056	-0.0052	0.0004	1.08
8	CH···O	0.0120	0.0242	0.0058	-0.0056	0.0002	1.03
9	CH···Ca	0.0105	0.0153	0.0040	-0.0041	-0.0001	0.96
10	CH···O	0.0133	0.0283	0.0068	-0.0066	0.0002	1.03
11	CH···O	0.0143	0.0369	0.0085	-0.0079	0.0006	1.08
12	CH···O	0.0199	0.0511	0.0127	-0.0126	0.0001	1.00
13	NH···O	0.0514	0.1246	0.0412	-0.0512	-0.0100	0.80
14	CH···O	0.0386	0.0961	0.0287	-0.0287	0.0006	1.00
15	Ca···O	0.0114	0.0229	0.0054	-0.0052	0.0003	1.04
16	Ca···O	0.0143	0.0448	0.0098	-0.0085	0.0013	1.15
17	O···O	0.0244	0.0705	0.0177	-0.0176	0.0001	1.01
18	CH···O	0.0152	0.0407	0.0094	-0.0087	0.0007	1.07

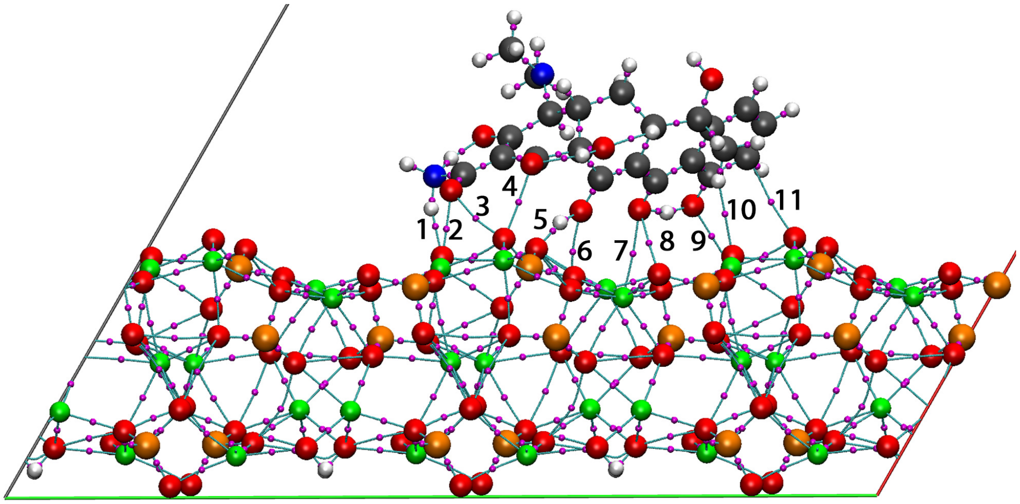

Figure S8: molecular graph including the bcp in magenta for configuration 1 in PO_4 surface for TC.

Table S4: qtaim parameters of the bcp's for the interactions between configuration 1 of tc and PO_4-ha.

interaction	$x_{ha} \cdots x_{mino}$	ρ(r)	$\nabla^2\rho$(r)	g_{bcp}	v_{bcp}	h_{bcp}	$-g_{bcp}/v_{bcp}$
1	NH···O	0.0574	0.1323	0.0466	-0.0601	-0.0135	0.77
2	Ca···O	0.0199	0.0813	0.0177	-0.0152	0.0025	1.16
3	O···O	0.0145	0.0466	0.0102	-0.0088	0.0014	1.15
4	O···O	0.0136	0.0551	0.0114	-0.0090	0.0023	1.25
5	OH···O	0.1148	0.1690	0.1060	-0.1698	-0.0637	0.62
6	O···O	0.0175	0.0528	0.0122	-0.0112	0.0010	1.09
7	Ca···O	0.0121	0.0313	0.0070	-0.0063	0.0007	1.12
8	O···O	0.0146	0.0502	0.0109	-0.0092	0.0016	1.18
9	Ca···O	0.0268	0.1249	0.0277	-0.0242	0.0034	1.14
10	CH···O	0.0108	0.0272	0.0060	-0.0053	0.0007	1.13
11	CH···O	0.0106	0.0289	0.0063	-0.0053	0.0009	1.17

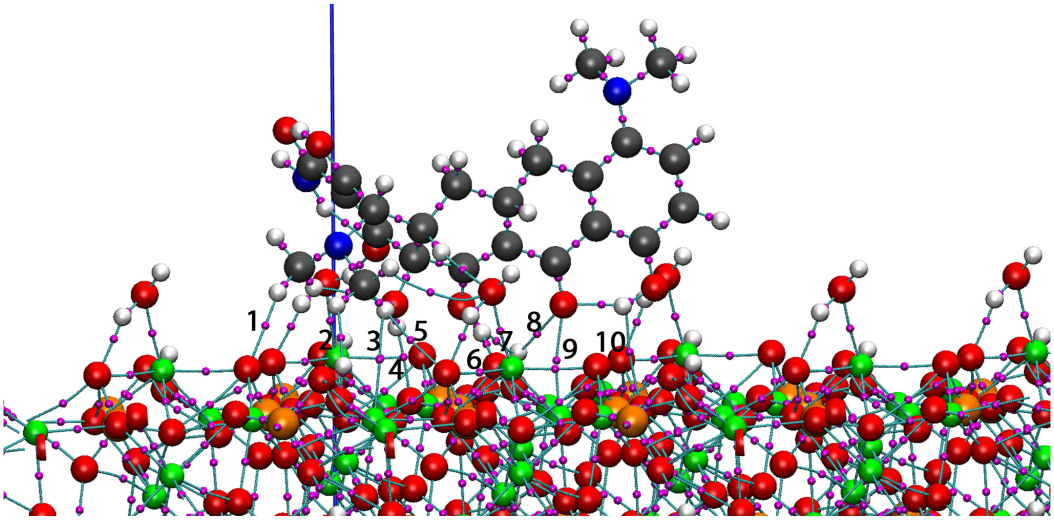

Figure S9: molecular graph including the bcp in magenta for configuration 1 in 001 surface for mino.

Table S5: qtaim parameters of the bcp's for the interactions between configuration 1 of mino and 001-ha.

interaction	$x_{ha} \cdots x_{mino}$	ρ(r)	$\nabla^2\rho$(r)	g_{bcp}	v_{bcp}	h_{bcp}	$-g_{bcp}/v_{bcp}$
1	CH3$\cdots$O	0.0150	0.0401	0.0093	-0.0086	0.0007	1.08
2	CH3$\cdots$HO	0.0117	0.0213	0.0053	-0.0053	0.0001	1.0
3	CH3$\cdots$Ca	0.0160	0.0429	0.0100	-0.0094	0.0006	1.06
4	OH$\cdots$O	0.0257	0.0681	0.0178	-0.0185	-0.0008	0.96
5	CH3$\cdots$O	0.0094	0.0211	0.0047	-0.0042	0.0006	1.12
6	OH$\cdots$O	0.0844	0.1664	0.0743	-0.1071	-0.0327	0.69
7	O$\cdots$HO	0.0196	0.0449	0.0116	-0.0119	-0.0004	0.97
8	O$\cdots$HO	0.0160	0.0429	0.0100	-0.0116	-0.0006	0.86
9	O$\cdots$HO	0.0089	0.0010	0.0028	-0.0030	-0.0003	0.93
10	OH$\cdots$O	0.0074	0.0181	0.0038	-0.0041	-0.0007	0.92

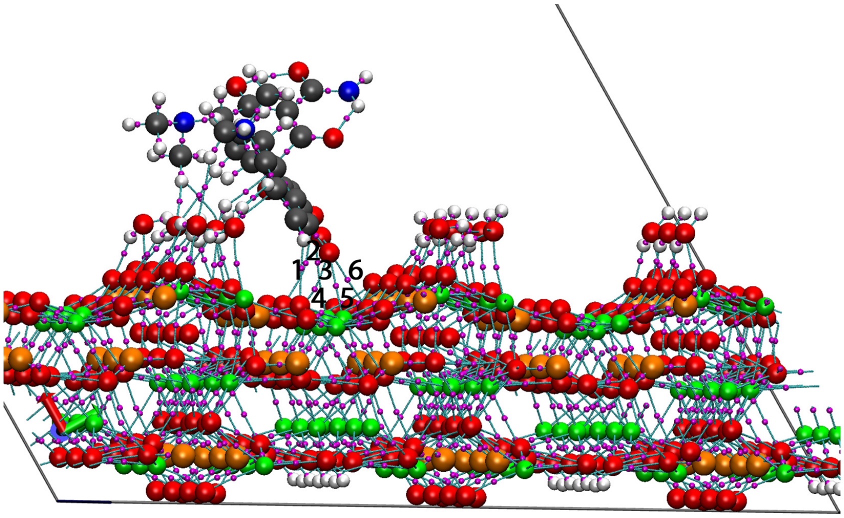

Figure S10: molecular graph including the bcp in magenta for configuration 1 in PO_4 surface for mino.

Table S6: qtaim parameters of the bcp's for the interactions between configuration 1 of mino and PO_4-ha.

interaction	$x_{ha} \cdots x_{mino}$	$\rho(r)$	$\nabla^2\rho(r)$	g_{bcp}	v_{bcp}	h_{bcp}	$-g_{bcp}/v_{bcp}$
1	CH$\cdots$O	0.0174	0.0384	0.0089	-0.0082	0.0007	1.08
2	O$\cdots$O	0.0039	0.0089	0.0018	-0.0013	0.0005	1.38
3	O$\cdots$Ca	0.0040	0.0048	0.0011	-0.0010	0.0001	1.1
4	O$\cdots$O	0.0136	0.0339	0.0079	-0.0073	0.0006	1.08
5	O$\cdots$Ca	0.0406	0.2040	0.0477	-0.0445	0.0033	1.07
6	O$\cdots$O	0.0163	0.0434	0.0102	-0.0096	0.0006	1.06

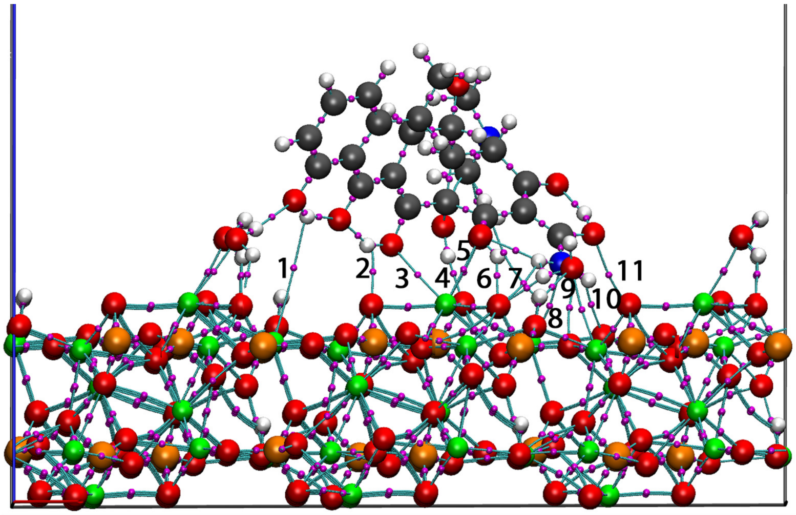

Figure S11: molecular graph including the bcp in magenta for configuration 2 in 001 surface for dox.

Table S7: qtaim parameters of the bcp's for the interactions between configuration 2 of dox and PO_4-ha.

interaction	$x_{ha} \cdots x_{mino}$	$\rho(r)$	$\nabla^2\rho(r)$	g_{bcp}	v_{bcp}	h_{bcp}	$-g_{bcp}/v_{bcp}$
1	OH···Ca	0.0025	0.0031	0.0007	-0.0005	0.0001	1.4
2	OH···O	0.0513	0.1249	0.0411	-0.0511	-0.0099	0.80
3	O···Ca	0.0142	0.0426	0.0095	-0.0083	0.0012	1.14
4	OH···O	0.0535	0.1265	0.0429	-0.0541	-0.0113	0.79
5	O···Ca	0.0268	0.1253	0.0278	-0.0242	0.0036	1.14
6	O···Ca	0.0250	0.0751	0.0187	-0.0185	0.0001	1.01
7	NH···O	0.0143	0.0351	0.0082	-0.0077	0.0005	1.06
8	N···Ca	0.0092	0.0136	0.0034	-0.0034	-0.0001	1.0
9	N···O	0.0105	0.0283	0.0062	-0.0052	0.0009	1.19
10	NH···O	0.0144	0.0386	0.0089	-0.0081	0.0008	1.09
11	O···O	0.0144	0.0352	0.0083	-0.0078	0.0005	1.06

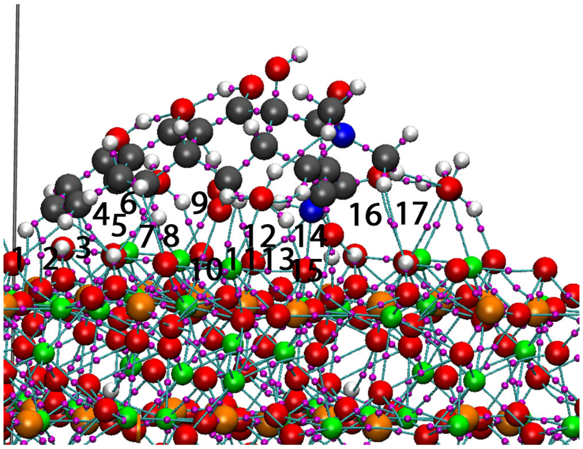

Figure S12: molecular graph including the bcp in magenta for configuration 2 in 001 surface for TC.

Table S8: qtaim parameters of the bcp's for the interactions between configuration 2 of TC and 001-ha.

interaction	$x_{ha} \cdots x_{mino}$	$\rho(r)$	$\nabla^2\rho(r)$	g_{bcp}	v_{bcp}	h_{bcp}	$-g_{bcp}/v_{bcp}$
1	CH$\cdots$O	0.0141	0.0365	0.0084	-0.0078	0.0007	1.07
2	CH$\cdots$O	0.0089	0.0239	0.0051	-0.0042	0.0009	1.21
3	CH$\cdots$OH	0.0189	0.0034	0.0095	-0.0105	-0.0010	0.90
4	CH$\cdots$O	0.0085	0.0232	0.0049	-0.0040	0.0009	1.22
5	CH$\cdots$OH	0.0237	0.0426	0.0127	-0.0148	-0.0021	0.85
6	CH$\cdots$Ca	0.0199	0.0497	0.0124	-0.0125	-0.0001	0.99
7	CH$\cdots$O	0.0198	0.0496	0.0124	-0.0125	-0.0001	0.99
8	CH$\cdots$O	0.0173	0.0466	0.0111	-0.0106	0.0005	1.04
9	O$\cdots$Ca	0.0266	0.1217	0.0271	-0.0238	0.0033	1.13
10	O$\cdots$Ca	0.0097	0.0188	0.0044	-0.0041	0.0003	1.07
11	CH$\cdots$O	0.0061	0.0152	0.0031	-0.0024	0.0007	1.29
12	OH$\cdots$O	0.0338	0.0916	0.0254	-0.0279	-0.0025	0.91
13	N$\cdots$O	0.0106	0.0026	0.0056	-0.0051	0.0007	1.09
14	N$\cdots$Ca	0.0125	0.0292	0.0068	-0.0063	0.0005	1.07
15	O$\cdots$O	0.0128	0.0445	0.0094	-0.0072	0.0017	1.30
16	O$\cdots$O	0.0114	0.0418	0.0086	-0.0068	0.0018	1.26
17	CH$\cdots$O	0.0045	0.0083	0.0017	-0.0014	0.0036	1.21

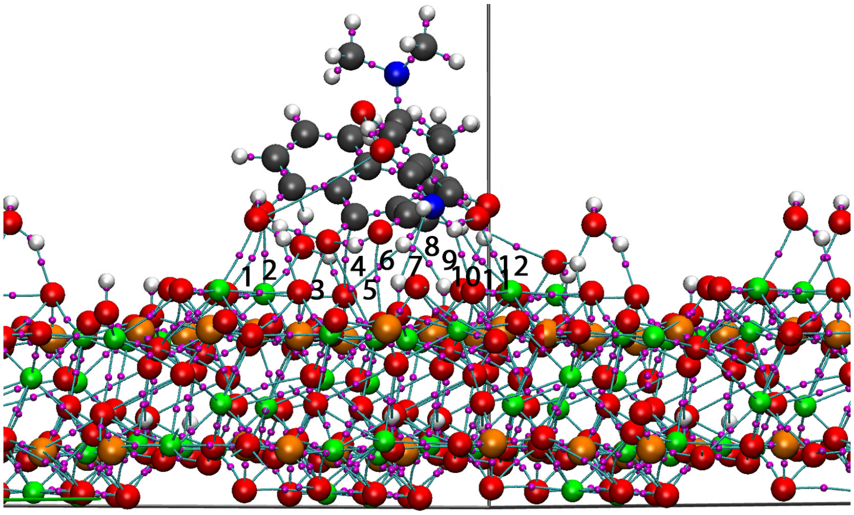

Figure S13: molecular graph including the bcp in magenta for configuration 1 in PO_4 surface for TC.

Table S9: qtaim parameters of the bcp's for the interactions between configuration 1 of TC and PO_4-ha.

interaction	$x_{ha}\cdots x_{mino}$	$\rho(r)$	$\nabla^2\rho(r)$	g_{bcp}	v_{bcp}	h_{bcp}	$-g_{bcp}/v_{bcp}$
1	O···O	0.0135	0.0357	0.0081	-0.0074	-0.0008	1.09
2	O···Ca	0.0173	0.0608	0.0135	-0.0117	0.0018	1.15
3	O···Ca	0.0083	0.0020	0.0043	-0.0036	0.0007	1.19
4	C···O	0.0092	0.0256	0.0054	-0.0044	0.0010	1.22
5	O···O	0.0139	0.0477	0.0103	-0.0086	0.0017	1.19
6	CH···O	0.0151	0.0398	0.0093	-0.0086	0.0007	1.08
7	N···HO	0.0101	0.0207	0.0048	-0.0045	0.0037	1.06
8	CH···HO	0.0201	0.0368	0.0104	-0.0116	-0.0012	0.89
9	NH···O	0.0308	0.0807	0.0122	-0.0241	-0.0020	0.50
10	OH···O	0.0457	0.1128	0.0356	-0.0430	-0.0074	0.82
11	CH···O	0.0075	0.0183	0.0039	-0.0032	0.0007	1.21
12	O···Ca	0.0267	0.1256	0.0278	-0.0241	0.0036	1.15
